# Increased Anxiety-Like Behavior in the Acute Phase of a Preclinical Model of Periodontal Disease

**DOI:** 10.3389/fneur.2020.598851

**Published:** 2020-12-22

**Authors:** Bruna Luiza Roim Varotto, Raquel Chacon Ruiz Martinez, Flavia Venetucci Gouveia, Geiza Fernanda Antunes, Gisele Maria de Campos Fabri, Gerson Ballester, Reynaldo Antequera, Silvia Regina Dowgan Tesseroli de Siqueira, Erich Talamoni Fonoff, Manoel Jacobsen Teixeira, José Tadeu Tesseroli de Siqueira

**Affiliations:** ^1^Dental Team, and Dental Research Group on Pain and Mental Health of Institute of Psychiatry, School of Medicine, University of São Paulo, São Paulo, Brazil; ^2^Division of Neuroscience, Hospital Sirio Libanês, São Paulo, Brazil; ^3^School of Medicine, LIM/23 – Institute of Psychiatry, University of São Paulo, São Paulo, Brazil; ^4^Biological Sciences Platform, Sunnybrook Research Institute, Toronto, ON, Canada; ^5^Clinical Dentistry Department, University of Juiz de Fora, Juiz de Fora, Brazil; ^6^School of Medicine, City of São Paulo University, São Paulo, Brazil; ^7^Pain Center and Division of Functional Neurosurgery, Department of Neurology, School of Medicine, University of São Paulo, São Paulo, Brazil; ^8^Orofacial Pain Team, Dentistry Division, School of Medicine, Hospital das Clinicas, University of São Paulo, São Paulo, Brazil

**Keywords:** nociception and pain, periodontal disease, anxiety, animal model, neuroplasticity

## Abstract

Periodontal disease (PD) is an infectious-inflammatory oral disease that is highly prevalent among adolescence and adulthood and can lead to chronic orofacial pain and be associated with anxiety, stress and depression. This study aimed to identify anxiety-like behaviors in the ligature-induced murine preclinical model of PD in different phases of the disease (i.e., acute vs. chronic). Also, we investigated orofacial mechanical allodynia thresholds and superficial cortical plasticity along the orofacial motor cortex in both disease phases. To this aim, 25 male Wistar rats were randomly allocated in acute (14 days) or chronic (28 days) ligature-induced-PD groups and further divided into active-PD or sham-PD. Anxiety-like behavior was evaluated using the elevated plus maze, mechanical allodynia assessed using the von Frey filaments test and superficial motor cortex mapping was performed with electrical transdural stimulation. We observed increased anxiety-like behavior in active-PD animals in the acute phase, characterized by decreased number of entries into the open arm extremities [*t*_(1,7)_ = 2.42, *p* = 0.04], and reduced time spent in the open arms [*t*_(1,7)_ = 3.56, *p* = 0.01] and in the open arm extremities [*t*_(1,7)_ = 2.75, *p* = 0.03]. There was also a reduction in the mechanical allodynia threshold in all active-PD animals [Acute: *t*_(1,7)_ = 8.81, *p* < 0.001; Chronic: *t*_(1,6)_ = 60.0, *p* < 0.001], that was positively correlated with anxiety-like behaviors in the acute group. No differences were observed in motor cortex mapping. Thus, our findings show the presence of anxiety-like behaviors in the acute phase of PD making this a suitable model to study the impact of anxiety in treatment response and treatment efficacy.

## Introduction

Periodontal disease (PD) is a highly prevalent chronic, infectious-inflammatory oral disease, responsible for early tooth loss, gingival bleeding and pain (1–3). It estimated that PD affects 20–50% of the global population, including adolescents, adults and seniors (4). PD is associated with depression, anxiety and stress and can lead to impoverished quality of life (5–7). Furthermore, disorders of the periodontal ligament can induce neuronal adaptations within the central nervous system, such as changes in neuronal excitability and synaptic plasticity (8, 9), which could lead to chronic and refractory craniofacial pain (1, 10, 11).

Preclinical murine models of PD are important tools for studying the pathophysiology of the disease and providing important insights for new therapies (12–14). A widely used model of PD involves the placement of ligatures in the gingival sulcus around the molar tooth (i.e., ligature-induced periodontitis model), resulting in bacteria infiltration, accumulation of biofilm and disrupting the gingival epithelium (13, 15, 16). Moreover, animal models provide the opportunity to investigate mechanisms of brain plasticity (17, 18) and distinct aspects of acute and chronic phases of diseases (19). Although increased anxiety-like behaviors have been reported in murine models of inflammatory pain (20) and trigeminal neuropathic pain (21), no study to date has evaluated the presence of anxiety-like behaviors in distinct phases of PD (i.e., acute vs. chronic).

To address this gap, in this study, we investigated anxiety-like behaviors in the acute and chronic phase of ligature-induced murine model of PD and evaluated the presence of mechanical allodynia and superficial cortical plasticity along the orofacial motor cortex on both phases of the disease.

## Materials and Methods

### Subjects

Twenty-five male Wistar rats (140–180 g) obtained from the animal facility of the Medical School were used in the study. Animals were housed in pairs in regular rat cages containing wood shavings (polypropylene; 40 × 34 × 17cm), with free access to food and water in a 12 h dark/light cycle (lights on at 07:00) with controlled ambient temperature (22 ± 2°C). All experiments were performed in compliance with the guidelines for ethical use of animals in research involving pain and nociception (22) and the recommendations of the Brazilian Society of Neuroscience and Behavior, which in turn are based on the US National Institutes of Health Guide for the Care and Use of Laboratory Animals. The study was reviewed and approved by the Ethics Committee of the Medical School of the University of São Paulo (protocol #380/12). Furthermore, the experiments were reported in accordance with the Animal Research Reporting of *in vivo* Experiments guidelines (ARRIVE; https://arriveguidelines.org/).

### Study Design

**Figure 1:**
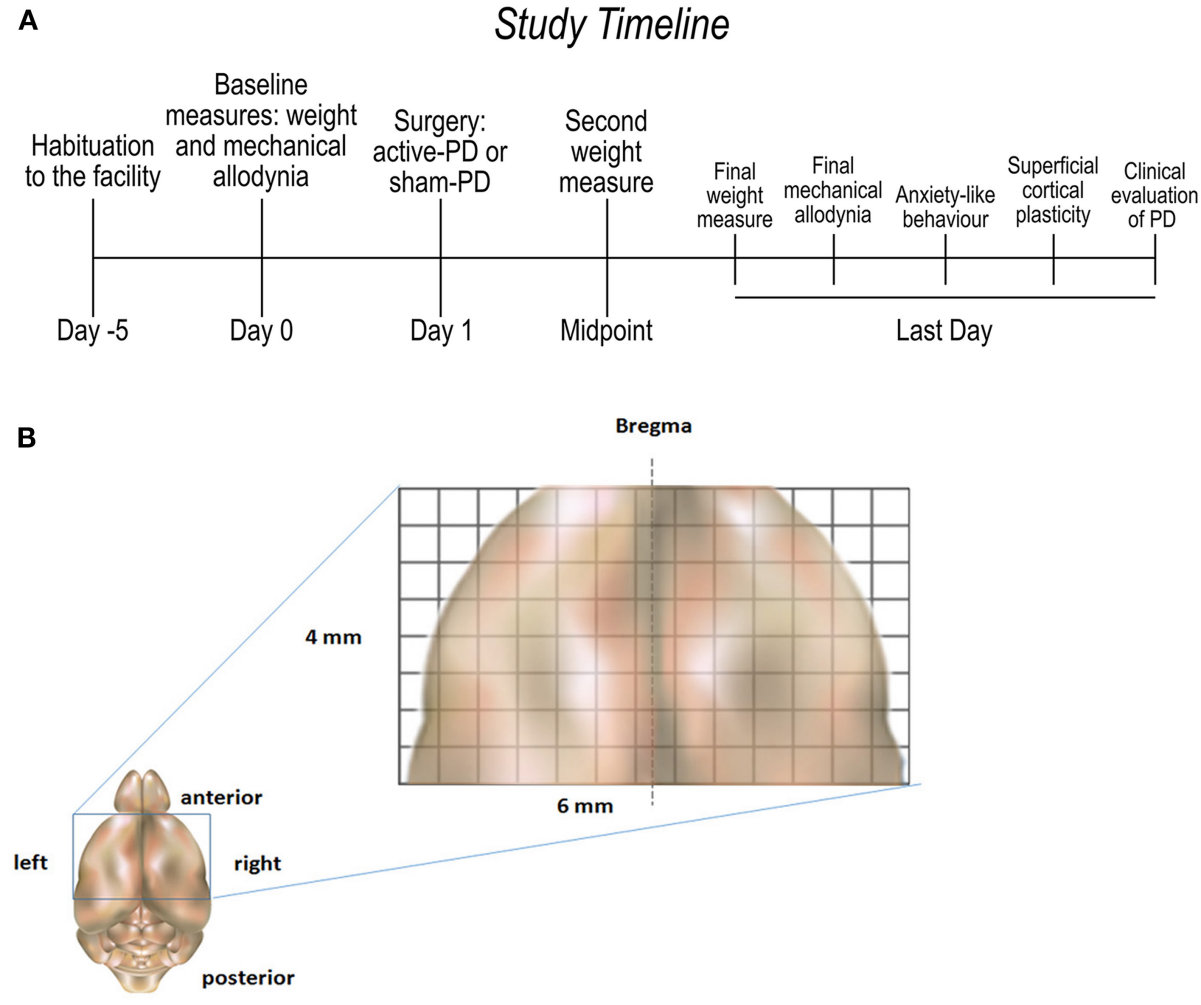
Study timeline **(A)** and illustration of the rat motor cortex area **(B)**. PD, periodontal disease; Midpoint, day 7 for Acute group and day 14 for Chronic group; Last day, Day 14 for Acute group and day 28 for Chronic group.

Following habituation to the animal facility, animals were allocated to groups Acute (14 days of PD) or Chronic (28 days of PD) and randomly assigned to activePD or shamPD (controls), resulting in 4 groups: (I) Acute-activePD (*n* = 7), (II) Acute-shamPD (*n* = 7), (III) Chronic-activePD (*n* = 6), (IV) Chronic-shamPD (*n* = 5). The experimental schedule was designed to have both groups at the same age during behavioral tests. Prior to receiving surgery, all animals were weighed and evaluated for a baseline measure of mechanical allodynia using von Frey filaments. On the following day, surgery was performed to induce activePD or shamPD (description follows), and animals were kept in the housing room for the number of days corresponding to the assigned group (i.e., 14 or 28 days). A second body weight measure was taken 7 (Acute group) or 14 (Chronic group) days after surgery. After the waiting period, animals were weighed and tests were performed to evaluate (I) final measure of mechanical allodynia (von Frey filaments), (II) anxiety-like behavior (elevated plus maze), and (III) the superficial cortical plasticity along the orofacial motor cortex (electrical transdural stimulation). See Figure 1A for study timeline.

### Surgery for Induction of Periodontal Disease

Animals were anesthetized with xylazine (50 mg/kg i.m.) and ketamine (100 mg/kg i.m.) and positioned on a surgical table designed for buccal cavity procedures. The condition of the gingiva was evaluated for exclusion of possible pre-existing diseases. After buccal and tongue retraction, a cotton ligature (4.0 Ethicon, Johnson & Johnson Company) was placed around the right mandibular first molar adjacent to the gingival margin, knotted on the mesio-buccal side and remained subgingival on the lingual side, as previously described (23, 24). On activePD animals the ligature remained in place throughout the experimental period (i.e., 14 or 28 days) and on shamPD animals the ligature was removed immediately after placement (23). The placement of a cotton ligature around the right mandibular first molar tooth induces PD by facilitating bacterial invasion of the gingival sulcus (25). The development of activePD was assessed clinically at the end of the experiments based on the description of the disease presented by Messer et al. (26).

### Body Weight Evaluation

Body weight was assessed by a blinded rater using a digital scale for three times throughout the study: (I) baseline measure before the surgical procedure, (II) second measure at midpoint (7 days for Acute group and 14 days for Chronic group), (III) third measure on the last day of experiment. All measures were taken on the 1st h of the light cycle.

### Evaluation of Anxiety-Like Behavior

The evaluation of anxiety behavior was performed using the elevated plus maze (EPM). This test comprises a maze elevated 50 cm above the ground, consisting of two closed arms and two open arms with a free central area that allows the animal to move through all spaces (27). Rats were placed at the junction of the four arms of the maze (the central area) with the nose facing one of the closed arms and were allowed to freely explore the apparatus for 5 min and the behavior was recorded for future analysis by a single blinded observer using X-Plot Rat 2005 1.1.0 software (FFCLRP-USP Laboratory of Prof. Silvio Morato de Carvalho, PhD). The apparatus was cleaned with a 5% ethanol solution and dried with a cloth between trials. Behavioral analysis was performed as previously described (28–30) and included the frequency of occurrence and total time spent on (I) open arms, (II) freezing (total absence of animal movement with the exception of respiration), (III) stretching (stretching the full length of the body with the forelimbs while keeping the hind limbs in place, and returning to the previous position), (IV) rearing (partial or total rising on the hind limbs), and (V) dipping (sticking the head outside the maze border and toward the floor).

### Mechanical Allodynia Threshold

Using a graded series of von Frey filaments (0.07–10 g–Touch Test Sensory Evaluator, CA, USA), mechanical allodynia thresholds were assessed on the ipsilateral whisker pad of all animals (activePD and shamPD), 1 day prior and 14 (Acute group) or 28 (Chronic group) days after surgery. Animals were transferred to the testing room 2 h before testing, and then individually placed in the experimental cage for a second habituation period of 10 min. A researcher blinded to group/condition performed the test, as previously described (31, 32). Briefly, animals were gently restrained with a cotton cloth and the von Frey filaments were applied in crescent order of force, with a 10 s interval between filaments. The smallest filament that elicited a back off/escape/attack reaction and/or head withdrawal in three consecutive applications was considered to be the mechanical allodynia threshold.

### Superficial Cortical Plasticity Along the Orofacial Motor Cortex

Active-PD and sham-PD animals of both Acute and Chronic groups were anesthetized with xylazine (50 mg/kg i.m.) and ketamine (100 mg/kg i.m.) and positioned on a stereotaxic apparatus (David Kopf Instruments, CA, USA). Local scalp injection of 2% lidocaine (1 ml/animal) was applied for local analgesia. A median axial incision was made in the scalp, followed by bilateral craniotomy above the motor cortex (4 × 6 mm, Figure 1B), using bregma as a reference point (33, 34). Bilateral mapping was performed via electrical transdural stimulation (1–15 volts) through a bipolar electrode with 200 μm between the tips (34, 35). The electrode was fixed to the stereotaxic bar and positioned in contact with the dura-mater. Stimuli were delivered with 500 μm between each other in all directions, covering the entire exposed area. An electrical stimulator (Grass 8,800–Grass Instruments, Quincy, MA, USA) produced the electrical stimuli that consisted of 1 s trains of 10 μs biphasic cathodal pulses delivered at 100 Hz. At each cortex point, the electrical stimuli were gradually increased up to a maximum of 15 volts and the animal's motor response was visualized in an upper side view. If no movements were seen until reaching the maximum voltage, the point was considered to be non-responsive. The same number of bilateral cortical points were evaluated in all animals. At the end of the experiment, for euthanasia, animals were deeply anesthetized with thiopental 150 mg/kg i.p. (Thiopentax, Cristalia).

### Statistical Analysis

Sample size calculation was performed based on the work of Meunier et al. (32) using a formula described previously (36), considering 5% of level of significance, 80% as power of the study, effect size of 12 and standard deviation of 1.5, resulting in a minimum of 4.2 rats per group. Data are reported as mean ± standard error of the mean (SEM). Statistical analysis was performed using SPSS Statistics 17.0 (IBM, 2008, USA). Mechanical allodynia threshold was evaluated as percentage of change from baseline measure ([last measure/first measure] ^*^ 100). Animals in the activePD group that presented reduced threshold and those in the shamPD group that maintained the threshold were included in the statistical analysis. The orofacial motor cortex was evaluated as the percentage of representation along the motor cortex. One animal of each group (*n* = 4) died during the cortical mapping procedure and were excluded from statistical analysis. Mechanical allodynia, anxiety-like behaviors (EPM) and superficial cortex mapping were analyzed using Student's *t*-test with independent measures, comparing activePD and shamPD animals. Body weight was analyzed using two-way repeated measures ANOVA [normal distribution Chi-Square = 3,42944, df = 1 (adjusted) *p* = 0,06404] and the Pearson correlation test was used for evaluating the correlation between mechanical allodynia and anxiety-like behaviors. The level of significance was set at *p* < 0.05 for all tests.

## Results

**Figure 2:**
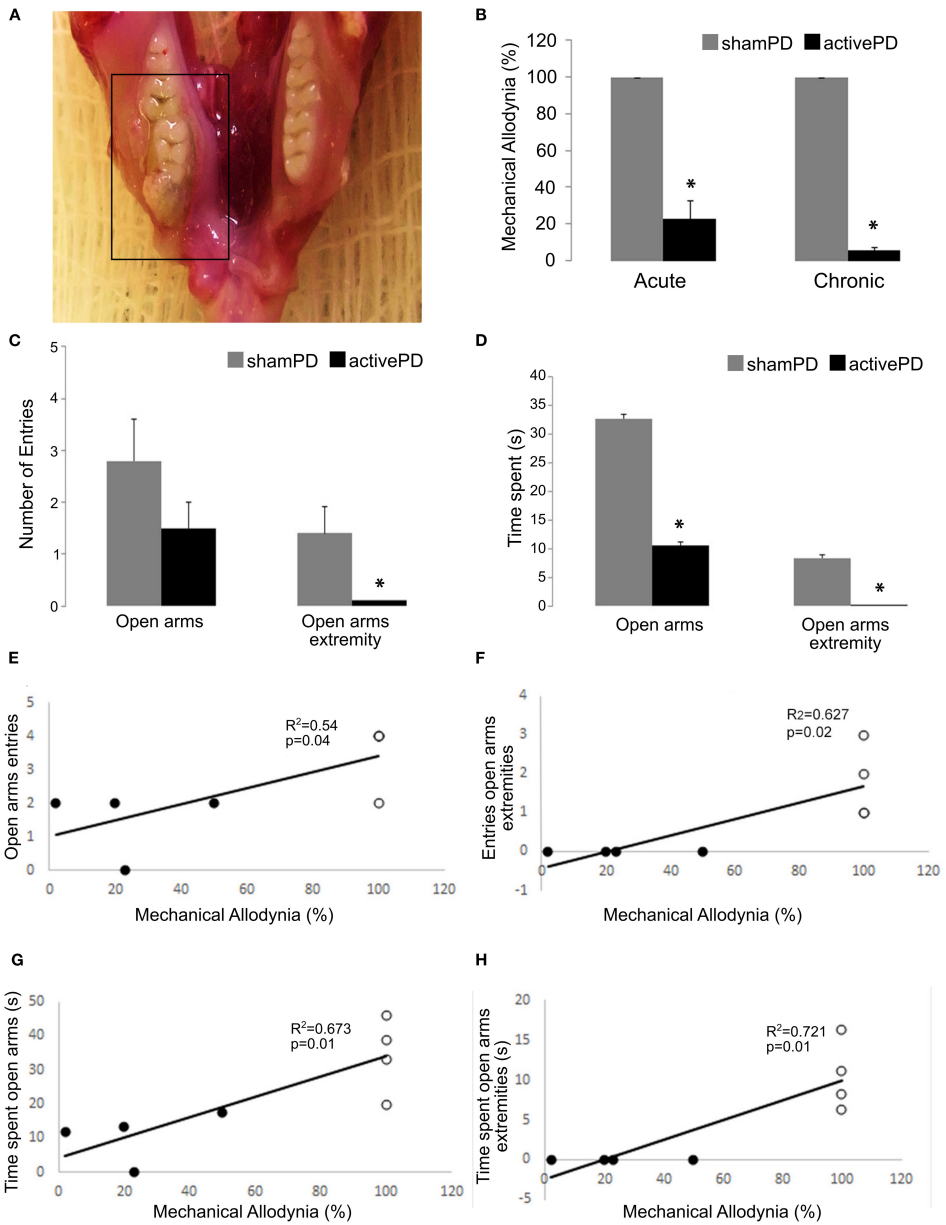
Illustrative case of moderate to severe active periodontal disease**. (A)** Reduction of mechanical allodynia threshold in Acute and Chronic groups at endpoint. **(B)** Increase in anxiety-like behaviors in the activePD animals compared to shamPD animals of the Acute group. **(C,D)** Positive correlation between mechanical allodynia thresholds and anxiety-like behaviors in the Acute group **(E–H)**. PD, periodontal disease. *indicates *p* < 0.05.

Clinical evaluation of the PD at endpoint showed all animals in the activePD groups (Acute and Chronic groups) presenting extensive ulceration of the gingiva with involvement of neighboring tooth, gingival inflammation, erythema, edema and accumulation of dental plaque, consisting with moderate to severe PD (Figure 2A) (26). No signs of PD were detected in shamPD animals of both Acute and Chronic groups. No differences in body weight were detected between activePD and shamPD animals [*Acute: F*_(4,64)_ = 0.033, *p* > 0.05; *Chronic: F*_(4,34)_ = 0.22, *p* > 0.05; Table 1]. There was a reduction in the mechanical allodynia threshold in the activePD groups compared to the shamPD groups at endpoint [*Acute: t*_(1,7)_ = 8.81, *p* < 0.001; *Chronic: t*_(1,6)_ = 60, *p* < 0.001; Figure 2B].

**Table 1:** Body weight measures (g).

**Body weight**	**Group 14**		**Group 28**	
	**ActivePD**	**ShamPD**	**ActivePD**	**ShamPD**
Measure 1	147 ± 10.85	150 ± 9.12	159 ± 13.38	163 ± 12.66
Measure 2	236 ± 13.25	242 ± 12.59	250 ± 17.96	268 ± 20.19
Measure 3	310 ± 23.67	312 ± 17.8	335 ± 22.83	353 ± 8.00

The EPM test showed an increase in anxiety-like behaviors in the activePD animals in the Acute group compared to shamPD animals of the same group. Specifically, the activePD group showed a decreased number of entries into the open arm extremities (Figure 2C) and reduced time spent in the open arms (Figure 2D) and open arm extremities (Figure 2D; Table 2). Also, there was a positive correlation between the mechanical allodynia threshold and anxiety-like behaviors in the Acute group (Figures 2E–H). No differences were observed in the remaining EPM parameters analyzed for the Acute group, as well as no differences in anxiety-like behaviors in animals of the Chronic group (Table 2). There were no differences observed between activePD and shamPD animals of both groups in the motor response elicited by superficial motor cortex electrical stimulation of the orofacial area, as well on areas that elicited no response (Table 3).

**Table 2:** Parameters measured in the Elevated Plus Maze (EPM) test.

**EPM parameter**	**Group 14**	**Group 28**
Open arms entries	*t*_(1,7)_ = 1.29, *p* = 0.24	*t*_(1,6)_ = 0.46, *p* = 0.66
Time spent in open arms	*t*_(1,7)_ = 3.56, *p* = 0.01	*t*_(1,6)_ = 0.80, *p* = 0.45
Open arm extremities entries	*t*_(1,7)_ = 2.42, *p* = 0.04	*t*_(1,6)_ = 0.68, *p* = 0.52
Time spent in open arm extremity	*t*_(1,7)_ = 2.75, *p* = 0.03	*t*_(1,6)_ = 0.67, *p* = 0.52
Crossing open arms	*t*_(1,7)_ = 1.87, *p* = 0.10	*t*_(1,6)_ = 0.49, *p* = 0.64
Stretching in open arms	*t*_(1,7)_ = 1.63, *p* = 0.14	*t*_(1,6)_ = 0.91, *p* = 0.39
Time stretching in open arms (s)	*t*_(1,7)_ = 1.75, *p* = 0.12	*t*_(1,6)_ = 0.48, *p* = 0.64
Time stretching in the center (s)	*t*_(1,7)_ = 1.10, *p* = 0.30	*t*_(1,6)_ = 0.68, *p* =0.51
Total stretching	*t*_(1,7)_ = 0.19, *p* = 0.84	*t*_(1,6)_ = 1.32, *p* = 0.23
Freezing in closed arms	*t*_(1,7)_ = 0.80, *p* = 0.44	*t*_(1,6)_ = 0.29, *p* = 0.77
Time freezing in closed arms (s)	*t*_(1,7)_ = 1.09, *p* = 0.30	*t*_(1,6)_ = 0.40, *p* = 0.70
Time dipping in the center (s)	*t*_(1,7)_ = 0.19, *p* = 0.84	*t*_(1,6)_ = 0.92, *p* = 0.38

**Table 3:** Percentage of representation of areas along the motor cortex.

**Cortical area**	**Group 14**			**Group 28**		
	**ActivePD**	**ShamPD**	***t*-test**	**ActivePD**	**ShamPD**	***t*-test**
Orofacial	20, 0.89	21, 0.68	*t*_(1,7)_ = 0.42, *p* = 0.68	22, 0.66	21, 0.44	*t*_(1,4)_ = 0.52, *p* = 0.63
Vibrissae	54.0, 9.90	68.0, 1.84	*t*_(1,7)_ = 1.58, *p* = 0.16	62.0, 8.88	65.0, 12.70	*t*_(1,4)_ = 0.19, *p* = 0.85
Mandible	8.0, 2.24	3.0, 1.10	*t*_(1,7)_ = 1.95, *p* = 0.09	6.0, 1.25	2.0, 1.14	*t*_(1,4)_ = 2.02, *p* = 0.11
Eye	4.0, 3.04	2.0, 1.05	*t*_(1,7)_ = 0.46, *p* = 0.66	1.0, 0.73	0.0, 0.00	*t*_(1,4)_ = 1.00, *p* = 0.37
Neck	8.0, 4.86	5.0, 1.59	*t*_(1,7)_ = 0.58, *p* = 0.58	5.0, 1.85	2.0, 2.33	*t*_(1,4)_ = 0.93, *p* = 0.40
Limb	7.0, 4.32	6.0, 3.80	*t*_(1,7)_ = 0.05, *p* = 0.95	4.0, 1.50	6.0, 2.32	*t*_(1,4)_ = 0.71, *p* = 0.52
No response	18.0, 3.88	16.0, 3.82	*t*_(1,7)_ = 0.46, *p* = 0.66	23.0, 8.39	24.0, 9.54	*t*_(1,4)_ = 0.09, *p* = 0.93

## Discussion

Improving the knowledge on the mechanisms of PD are fundamental to develop new therapies and improve treatment efficacy. Preclinical models can provide important insights into the pathophysiology of the disease, thus a better characterization of the behavioral phenotype of these animals is necessary. The ligature-induced model of PD used in this study can be divided into two distinct phases: acute and chronic (19). While the acute phase (≤14 days) is characterized by significant bone loss, pronounced inflammation of the affected region and elevated gene expression of pro-inflammatory cytokines, the chronic phase (>14 days) shows no further progression of bone loss and a constant state of inflammation (19). Thus, the presence of inflammatory signs (e.g., of gingival growth due to edema, erythema, and areas of ulceration) are clear signs of installed PD and the intensity of the symptoms can determine the severity of the disease (26). In our study, all animals allocated in the active-PD subgroup of both Acute and Chronic groups presented moderate to severe PD at the endpoint, showing the feasibility of this model to investigate both phases of the disease.

A growing problem among patients with PD is the presence of associated anxiety traits that can lead to treatment interruption, reduced treatment efficacy and aggravation of the severity of the disease (7, 37–39). We observed increased anxiety-like behaviors in active-PD animals of the Acute phase when comparing to sham-PD animals of the same group and a positive association between anxiety and mechanical allodynia of the affected orofacial region. Although mechanical allodynia is commonly observed in patients with different stages of periodontitis (40, 41), preclinical models of periodontitis show discordant results (42, 43). These discrepancies may be due to the technique employed to assess the mechanical allodynia threshold (e.g., sedated vs. awake animals) and the murine model used (e.g., mouse vs. rat). It is known that host susceptibility is a crucial factor for the development of periodontitis (44) resulting in great variability of clinical features between studies.

The presence of anxiety-like behaviors in models of trigeminal neuropathic pain (21) and inflammatory pain (20, 45) have been described in the literature and are thought to be related to mechanisms of neuroinflammation such as glial cell activation, increase in pro-inflammatory cytokines and infiltration of leukocytes (46). Albeit our study did not aim to evaluate these markers of neuroinflammation, it is plausible to assume these same factors could be influencing anxiety-like behaviors in the ligature-induced PD model as well. To investigate possible superficial cortical plasticity that could explain the behavioral differences observed in this study, we applied electrical transdural stimulation on specific areas of the motor cortex and observed the motor response generated (35). This technique allows for the functional mapping of the surface of the neocortex by evoking motor responses on specific body segments according to the coordinates used (35). Although we did not find significant results in our study, it is possible to assume that the use of a more refined technique for the detection of muscle activity (e.g., electromyography) while mapping the surface of the orofacial motor cortex could result in distinct outcomes.

Altogether, our results show that the ligature-induced PD in the acute phase is a suitable model for the study of anxiety-like behaviors in periodontitis, thus allowing for the investigation of the possible impacts of anxiety on the individual response to treatment and general treatment efficacy.

## Data Availability Statement

The data generated and analyzed during the current study are available from the corresponding author upon reasonable request.

## Ethics Statement

This study has the approval of the Ethics Committee of the Medical School of the University of São Paulo (protocol number: 380/12).

## Author Contributions

BV, RM, and FG performed experiments, analyzed the data, and wrote the draft manuscript. GF, GB, RA, and GA performed experiments and analyzed the data. RA, SS, MT, EF, and JS designed the study, analyzed the data, and wrote the draft manuscript. All authors contributed to manuscript revision and approved the final version.

## Conflict of Interest

The authors declare that the research was conducted in the absence of any commercial or financial relationships that could be construed as a potential conflict of interest.
